# A retrospective review of antibiotic use for acute respiratory infections in urgent-care patients

**DOI:** 10.1017/ash.2022.337

**Published:** 2022-12-05

**Authors:** Richard C. Childers, Bryan Bisanz, Gary M. Vilke, Jesse J. Brennan, Alexandrea O. Cronin, Edward M. Castillo

**Affiliations:** Department of Emergency Medicine, University of California–San Diego, San Diego, California

## Abstract

**Objective:**

We examined the use of antibiotics for acute respiratory infections in an urgent-care setting.

**Design:**

Retrospective database review.

**Setting:**

The study was conducted in 2 urgent-care clinics staffed by academic emergency physicians in San Diego, California.

**Patients:**

Visits for acute respiratory infections were identified based on presenting complaints.

**Methods:**

The primary outcome was a discharge prescription for an antibiotic. The patient and provider characteristics that predicted this outcome were analyzed using logistic regression. The variation in antibiotic prescriptions between providers was also analyzed.

**Results:**

In total, 15,160 visits were analyzed. The patient characteristics were not predictive of antibiotic treatment. Physicians were more likely than advanced practice practitioners to prescribe antibiotics (1.31; 95% confidence interval [CI], 1.21–1.42). For every year of seniority, a provider was 1.03 (95% CI, 1.02–1.03) more likely to prescribe an antibiotic. Although the providers saw similar patients, we detected significant variation in the antibiotic prescription rate between providers: the mean antibiotic prescription rate within the top quartile was 54.3% and the mean rate in the bottom quartile was 21.7%.

**Conclusions:**

The patient and provider characteristics we examined were either not predictive or were only weakly predictive of receiving an antibiotic prescription for acute respiratory infection. However, we detected a marked variation between providers in the rate of antibiotic prescription. Provider differences, not patient differences, drive variations in antibiotic prescriptions. Stewardship efforts may be more effective if directed at providers rather than patients.

The rapid growth of antibiotic-resistant infections is a significant public health problem; curbing unnecessary use of antibiotics in the healthcare setting is one part of the solution.^1,2^ The treatment of acute respiratory infections (including sinusitis, otitis media, pharyngitis, bronchitis, influenza, and viral and nonviral pneumonia) is the most common reason for antibiotic prescriptions in the outpatient setting.^3,4^ Systematic reviews have revealed that antibiotics provide little to no symptomatic relief for the common cold, acute otitis media in children, maxillary sinusitis, sore throat, and acute bronchitis.^5–9^ The number needed to prevent rare complications of acute respiratory infections (ARIs), such as mastoiditis or pneumonia, is >4,000 cases.^10^ However, it has been estimated that antibiotics are prescribed in more than one-half of outpatient visits for ARI.^4^ This is unfortunate because antibiotics are associated with diarrhea, allergic skin reactions, anaphylaxis, and the development of resistance in the individual taking the antibiotic.^11–14^

Understanding the characteristics of ARI antibiotic practices can inform efforts to reduce their unnecessary use. Previous studies have generally relied on large outpatient survey databases.^3,15–20^ There has been a large increase in the use of retail and urgent-care clinics, where ARIs are frequently seen. However, the antibiotic utilization patterns in this setting have not been specifically examined.^21,22^

In this retrospective review, we examined the use of antibiotics for ARI in patients in the urgent-care setting. We also investigated specific predictors of antibiotic use not available for study in large databases (including provider sex, seniority, and provider type) and the variation between provider antibiotic prescription rates.

## Methods

### Study setting and population

The study setting consisted of 2 urgent-care centers serving suburban populations associated with an academic healthcare system. The combined annual census comprises ∼20,000–30,000 visits. The providers included emergency physicians (EPs), nurse practitioners (NPs), and physician assistants (PAs). NPs and PAs were combined in the analysis and are referred to as advanced practice practitioners (APP). In these urgent-care centers, physicians and APPs generally encounter the same types of patients. No physician or APP trainees worked at these sites. Data on cases between September 2016 and November 2019 were obtained from electronic medical records. All ARI-presenting complaints were taken from the 50 most common complaints, including congestion, cough, ear pain, fever, flu-like symptoms, pharyngitis, sinus problems, sore throat, and throat pain. Cases with a secondary chief complaint were also excluded; these were often found to have confounders, such as a chief complaint of “UTI.” Cases with missing data were excluded.

### Primary outcome

The primary outcome was the presence of a discharge prescription for antibiotics in patients presenting to a urgent-care clinic with an ARI presenting complaint of ARI. A review of the most common ARI antibiotics was obtained from the *Sanford Guide to Antimicrobial Therapy*.^23^ Antibiotics included amoxicillin, amoxicillin-clavulanic acid, azithromycin, cefdinir, clindamycin, penicillin G, and penicillin V. We excluded other antibiotics, such as doxycycline and fluroquinolones, that theoretically could be used for ARI. In a preliminary review of our data, the incidence of their use was small and was often associated with non-ARI use, including UTI and cellulitis. Thus, these were excluded to minimize error.

Predictors of discharge prescription were determined a priori. They included both patient and provider characteristics. Patient characteristics included age, sex, and whether the patient was there for a repeat visit, which was defined as a visit within 2 weeks of the initial visit (both visits had to be for an ARI).

Provider characteristics included years since training graduation (physician residency, NP, or PA school), sex, provider type (physician vs APP), and whether the provider was a high prescriber of antibiotics. Providers were ranked according to the rate at which an antibiotic was prescribed for ARI visits: a high-prescribing provider was defined as above the 75th percentile and low prescribers were below the 25th percentile. Years since training graduation were obtained from departmental records, direct inquiry by e-mail, or through the website Doximity.^24^ In the provider analysis, those providers with <30 patient visits were excluded to decrease random error. This study was reviewed and approved by the local institutional review board.

### Data analysis

Patient, visit, and provider characteristics are also reported. The 2 most common antibiotics were assessed in each complaint group. Predictive factors for antibiotic prescription were assessed using unadjusted (single predictor) and adjusted (multipredictor) logistic regression. Effect sizes were summarized using odds ratios and 95% confidence intervals (CIs). No predictors were prespecified. The final multipredictor model was determined using backward model selection based on the Wald test with a threshold of inclusion of 0.20. Univariable results for high antibiotic prescribers were included as a reference but were not included in the final model because there was a correlation with the other variables.

Variations in the rate of antibiotic prescription for ARIs among urgent-care providers who saw at least 30 patients are also reported. The coefficient of variation (COV) was used to quantify variations, defined as the standard deviation divided by the mean value. These values were generated to quantify the variation among providers in terms of the antibiotic prescription rate. Patients with ARI are seen by providers in urgent-care settings in a somewhat random manner; this suggests that any variation seen is not driven by patient factors. To confirm this, age and presenting complaints were compared between high and low antibiotic prescribers and between physicians and APPs. Analyses were performed using SPSS for Mac version 26 software (IBM, Armonk, NY).

## Results

In total, 19,549 patients with the 50 most common chief complaints were identified as having ARIs between 2017 and 2019. We excluded 1,956 cases who had a second chief complaint and 2,433 cases with missing data, leaving 15,160 cases for analysis. Patient, provider, and visit characteristics are listed in Table 1. Approximately one-third of patients received antibiotics. Patients presenting with sinus problems had the highest antibiotic prescription rate (60%), and those with flu-like symptoms had the lowest rate (18.3%). Repeated visits represented 4.4% of all visits; 39.6% of patients who did not initially receive an antibiotic received one at the repeated visit. Notably, 21.0% of patients with repeated visits received an antibiotic prescription at both visits. Table 2 describes the prevalence of the 2 most common antibiotics for each presenting complaint group: azithromycin was the most common, followed by amoxicillin-clavulanic acid.

**Table 1.** Patient, Visit, and Provider Characteristics^a^

Patient Characteristic	Total
Patient mean age (SD)	40.7 (20.5)
Sex, male, %	38.3
Pediatric patients (aged <18 y), %	8.5
**Visit characteristics (n = 15,160), %**	
Encounters seen by advanced practice practitioners	34.9
Encounters seen by male providers	56.6
Encounters received antibiotics	35.3
Repeated visits^b^	4.4
No antibiotic first visit/yes antibiotic repeated visit	39.6
**Presenting complaint: % of visits (% received antibiotic)**	
Congestion	4.3 (39.0)
Cough	40.5 (33.3)
Ear pain	11.4 (41.7)
Fever	2.4 (24.3)
Flu-like symptoms	7.1 (18.3)
Sinus problem	8.4 (60.2)
Sore throat	25.8 (32.4)
**Provider characteristics (n = 81)**	
Advanced practice practitioners, %	30.7
Sex, male, %	49.5
Years since training completion, y (SD)	9.4 (8.5)

Note. SD, standard deviation; ARI, acute respiratory infection.

a Percentages may not total 100 because of rounding.

b Repeated visits defined as a visit for ARI complaint within 2 weeks of initial visit.

**Table 2.** The Two Most Common Antibiotics for Each Presenting Complaint Group

Presenting Complaint	Antibiotic (%)	Antibiotic (%)
Congestion	Azithromycin (62.5)	Amoxicillin-clavulanic acid (22.0)
Cough	Azithromycin (80.4)	Penicillin (11.9)
Ear pain	Azithromycin (62.5)	Amoxicillin-clavulanic acid (22.0)
Fever	Azithromycin (38.9)	Amoxicillin (30.5)
Sinus problem	Amoxicillin-clavulanic acid (46.1)	Azithromycin (37.9)
Flu-like symptoms	Azithromycin (63.0)	Amoxicillin-clavulanic acid (15.3)
Sore throat	Azithromycin (28.7)	Penicillin (27.7)

Table 3 illustrates the results of the logistic regression model that determines the predictors of receiving an antibiotic prescription; only provider type and seniority were included in the final model. The patient-associated factors were not statistically significant. In univariable analysis, being seen by a physician (instead of an APP), a male provider or a senior provider were all associated with increased odds of receiving an antibiotic; however, provider sex was not significant in the multivariable model. Provider type and seniority were statistically significant predictors. Unsurprisingly, being seen by a high antibiotic prescriber was associated with a 2.9 times higher likelihood of receiving an antibiotic.

**Table 3.** Predictors of Receiving an Antibiotic for Acute Respiratory Infections Assessed Using Unadjusted (Single-Predictor) and Adjusted (Multipredictor) Logistic Regression Analysis^a^

Predictor	Univariable OR (95% CI)	*P* Value	Multivariable OR (95% CI)	*P* Value
**Patient characteristics**				
Age, y	1.0 (.99–1.00)	.82	Not included	
Sex, male	1.05 (.98–1.13)	.12	Not included	
Repeated visit^b^	0.96 (.81–1.13)	.62	Not included	
**Provider characteristics**				
Physician (vs advanced practice practitioner)	1.57 (1.46–1.69)	<.001	1.31 (1.21–1.42)	<.001
Sex, male	1.38 (1.29–1.48)	<.001	Not included	
Years since training graduation	1.03 (1.03–1.04)	<.001	1.03 (1.02–1.03)	<.001
High antibiotic prescriber^c^	2.91 (2.70–3.14)	<.001	Not included	

Note. OR, odds ratio; CI, confidence interval.

a The final multivariable model was determined using backward model selection with a 0.20 threshold for inclusion.

b Repeated visit was defined as a visit for an acute respiratory complaint within 2 weeks of initial visit.

c High antibiotic prescriber was defined as being in the top quartile of antibiotic prescribers. It was included as a reference in the univariate analysis, but not in the multivariable analysis, as it was associated with the other predictors.

**Fig. 1.**
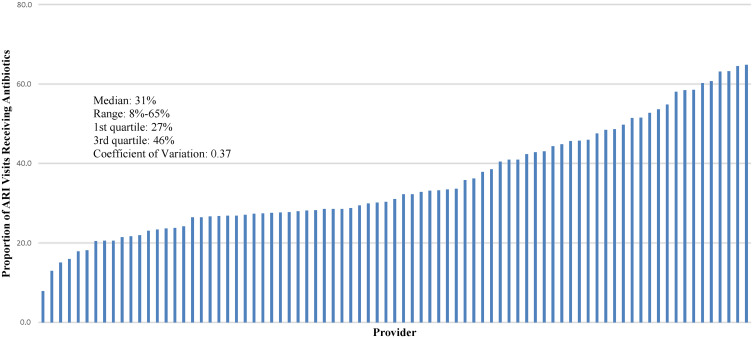
Variation in the rate of antibiotic prescription for acute respiratory infections in urgent-care providers who saw at least 30 patients.

Figure 1 illustrates the variation in antibiotic prescription rates. Prescription rate was defined as the number of visits that a specific provider prescribed an antibiotic for an ARI divided by the total number of ARI visits. The mean antibiotic prescription rate within the top quartile was 54.3%, whereas the mean rate in the bottom quartile was 21.7%, with a range of 8%–65%. The coefficient of variation was 0.37; for reference, the 2005 coefficient of variation in state-level Medicare spending per beneficiary, considered large, was 0.11.^25^

To determine whether patient-level factors were driving this variation, the age and chief-complaint distribution are compared between high and low prescribers, as well as between APPs and physicians (Table 4). No significant differences were found between the groups. This finding is consistent with provider, not patient, factors driving the large variation in antibiotic prescription rates.

**Table 4.** A Comparison of the Patient Characteristics Between High and Low Antibiotic Prescribers, and Between Advanced Practice Practitioners (APP) and Physicians^a^

	High Prescribers	Low Prescribers
Age, mean y (SD)	41.8 (20.1)	40.3 (20.5)
**Presenting complaint, %**		
Congestion	4.5	4.2
Cough	41.7	40.1
Ear pain	10.7	11.6
Fever	2.6	2.4
Flu-like symptoms	6.4	7.4
Sinus problem	9.4	8.1
Sore throat	24.6	26.2
	APPs	Physicians
Age, mean y (SD)	38.5 (20.1)	41.9 (20.7)
**Presenting complaint, %**		
Congestion	4.0	4.5
Cough	38.8	41.4
Ear pain	11.8	11.1
Fever	2.8	2.2
Flu-like symptoms	7.5	6.9
Sinus problem	7.7	8.8
Sore throat	27.4	28.5

Note. SD, standard deviation.

a A high prescriber provider was above the 75th percentile in utilization of antibiotics, and low prescribers were below the 25th percentile.

This study had several limitations. This multisite study within a single health system may not have external validity in other practice settings or existing literature. The 2 urgent-care clinics in our study, associated with an academic medical center in southern California, serve a suburban population. The Western region generally has a lower rate of antibiotic prescription compared to the Northeast, South, and Midwest regions.^3,20,26–28^ As of 2018, 78% of urgent-care clinics are located in suburban areas.^29^ It is not clear how the association with an academic medical center would affect results. The literature on antibiotic use for ARI generally relies on large national surveys, such as the National Ambulatory Medical Care Survey; this should be considered when comparing results to our study.

One benefit of this single-center study was access to variables that are infrequently studied, such as provider sex, seniority, and professional degree. However, the numbers were small for certain categories of providers, and we were unable to include these variables in the multivariable analysis. We did not find that a repeated visit for an ARI predicted receiving an antibiotic prescription; however, this result was limited to repeated visits within our system because visits outside our system were not captured.

## Discussion

In this study of antibiotic use for ARI in urgent-care patients, the rate of antibiotic use was lower than that generally found in the literature. The overall antibiotic prescription rate for ARIs is ∼60%; we recorded a rate of 35%.^4,17^ The rates for bronchitis and pharyngitis were both ∼60%; we recorded rates of 33% and 32.4%, respectively,.^3,16,17,20,26^ We found less of a difference for sinusitis patients: nationally, the rate is ∼70%, and we recorded a rate of 60%.^3,17^ The lower antibiotic rate could be due to differences in patient population, study methodology, or chance. An alternative hypothesis could be the association of this urgent-care center with an academic medical center, which may be worthy of further study.

In general, neither patient age nor sex has been found to predict antibiotic prescription for ARI.^17,20,26,27,30–32^ This was the case in the present study. We did not study patient race; again, with some exceptions, this factor is generally weakly predictive of receiving an antibiotic for ARI.^17,20,26,27,30^

In this study, provider sex did not predict prescribing an antibiotic, but older providers and physicians (compared to APPs) were more likely to provide a prescription. However, the existing literature examining provider characteristics is inconsistent. McKay et al (2019)^32^ discovered that older male providers were more likely to prescribe antibiotics, whereas Jones et al (2015)^31^ noted no difference. Suda et al (2016)^33^ reported that APPs were increasing their rate of prescribing antibiotics while physicians were decreasing their rate; however, physicians still prescribed antibiotics at a higher rate.^33^ Shaver et al (2019)^27^ also determined that patients seen by APPs were less likely to receive antibiotics. However, Frost et al (2018)^34^ found that APPs prescribe more, and Jones et al (2015)^31^ reported no difference between physicians and APPs.

In summary, we did not detect consistent identifiable patient or provider characteristics that can predict antibiotic prescriptions for ARIs. In our analysis, provider characteristics were more predictive than patient characteristics but were still only weakly predictive.

Variation in the rate of antibiotic prescription for ARIs has been well established.^27,31,32,34,35^ Variation was measured between different variables, including geographic regions, insurance plans, and specialties. However, only 2 studies have recorded variations in ARI treatment between providers, both of which were drawn from large databases.^31,32^ We discovered that significant variation is not surprising, but it is novel because we present it in the urgent-care population specifically, and it was among providers seeing the same population of patients.

Antibiotic stewardship programs can decrease antibiotic use.^36^ However, persistently high rates of overuse suggest that there is still work to be done. Our data, showing high variation in rates of antibiotic utilization among providers seeing the same types of patients, suggest that efforts directed toward providers, not patients, might be more effective.
